# Atypical Nodular Hidradenoma of the Postauricular Region: A Report of a Rare Case With Radiological, Histopathological, and Immunohistochemical Correlation

**DOI:** 10.7759/cureus.114461

**Published:** 2026-08-13

**Authors:** Raj P Chaudhary, Ajeet K Khilnani, Mahalaxmi Pillai, Shweta Sardhara, Pragati Bharai

**Affiliations:** 1 Otolaryngology - Head and Neck Surgery, Gujarat Adani Institute of Medical Sciences, Bhuj, IND; 2 Plastic and Reconstructive Surgery, Gujarat Adani Institute of Medical Sciences, Bhuj, IND

**Keywords:** adnexal tumor, atypical nodular hidradenoma, immunohistochemistry (ihc), post-auricular swelling, sweat gland tumor

## Abstract

Atypical nodular hidradenoma is an uncommon adnexal neoplasm arising from eccrine sweat glands, with histological features intermediate between benign hidradenoma and hidradenocarcinoma. These tumors are rarely encountered in the head and neck region, particularly in the postauricular area, posing significant diagnostic challenges because of their nonspecific clinical and radiological appearance. We report the case of a 55-year-old male presenting with a slowly progressive postauricular swelling of four years’ duration. Imaging demonstrated a well-defined, enhancing subcutaneous lesion without underlying bone or muscle invasion. Fine-needle aspiration cytology suggested a benign adnexal neoplasm. The patient underwent complete surgical excision with local flap reconstruction. Histopathological examination revealed an atypical adnexal tumor characterized by solid and cystic architecture, a dual cell population, focal infiltrative margins, and an absence of overt malignant features. Immunohistochemistry demonstrated a low proliferative index (Ki-67 approximately 1%), focal smooth muscle actin positivity, patchy S100 positivity, and negative p63 staining, confirming the diagnosis of atypical nodular hidradenoma. The postoperative period was uneventful, with no evidence of recurrence during follow-up. This report highlights the importance of complete surgical excision, histopathological examination, and immunohistochemistry in establishing the diagnosis and guiding management of this rare tumor.

## Introduction

Nodular hidradenoma is a benign adnexal neoplasm originating from eccrine sweat glands and is characterized by solid and cystic differentiation. Because of its infrequency, exact global prevalence statistics are poorly documented, although the annual incidence rate for all sweat gland tumors is extremely low, at approximately 5.1 cases per one million people. Although these tumors may occur throughout the body, they are most frequently found on the trunk and extremities, while involvement of the head and neck remains uncommon [1]. Their clinical presentation is usually nonspecific, consisting of a slow-growing, painless dermal or subcutaneous nodule, making preoperative diagnosis difficult.

Atypical nodular hidradenoma represents a histological variant demonstrating increased architectural complexity and focal infiltrative growth without fulfilling the criteria for hidradenocarcinoma. Because of its rarity, especially in the postauricular region, there is limited literature regarding its biological behavior, optimal management, and prognostic significance [2].

Radiological findings are often nonspecific, and cytological diagnosis may overlap with other adnexal tumors. Therefore, histopathological examination supplemented with immunohistochemistry remains the gold standard for definitive diagnosis. Complete surgical excision with clear margins is recommended because incomplete removal has been associated with local recurrence [3].

We report a rare case of atypical nodular hidradenoma arising in the postauricular region, with emphasis on the clinical presentation, radiological findings, histopathological features, immunohistochemical profile, surgical management, and review of the available literature.

## Case presentation

A 55-year-old man presented to the Department of Otorhinolaryngology with a gradually enlarging swelling over the right postauricular region of four years’ duration. The swelling had an insidious onset and increased progressively without associated pain, bleeding, discharge, fever, or constitutional symptoms. The patient had a history of hypertension under medical treatment and had completed treatment for pulmonary tuberculosis approximately 36 years earlier. A biopsy performed four years previously had suggested either a proliferative pilar tumor or atypical nodular hidradenoma, but no definitive treatment had been undertaken.

**Figure 1:**
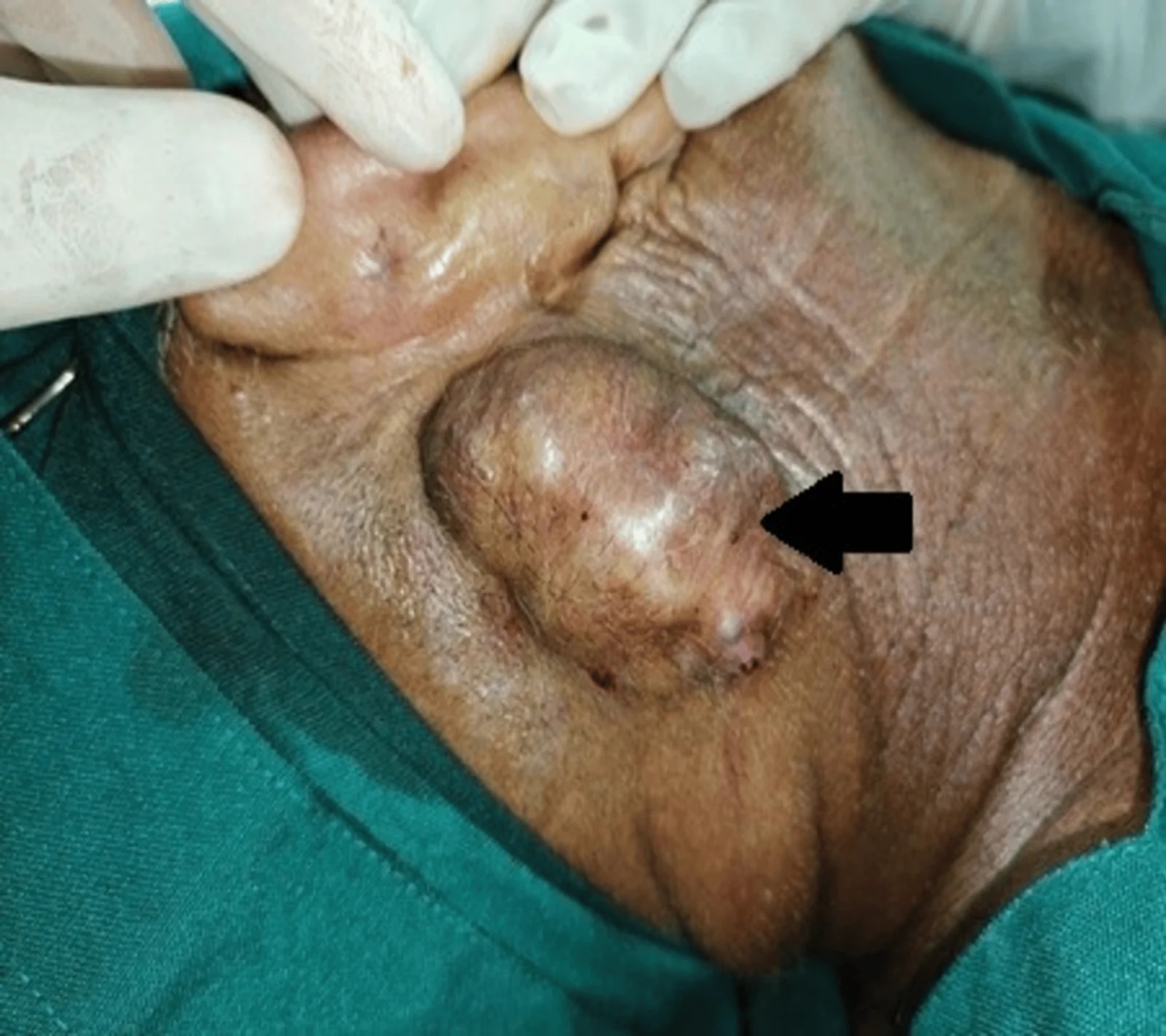
Preoperative clinical photograph showing a well-circumscribed right postauricular swelling (black arrow).

On examination, a well-defined, globular swelling measuring approximately 3 × 2 cm was present over the right postauricular region. The overlying skin appeared normal without ulceration or erythema. The swelling was firm, nontender, nonmobile, nonfluctuant, noncompressible, and nontransilluminant. There was no bruit on auscultation, and regional lymph nodes were not palpable (Figure 1).

**Figure 2:**
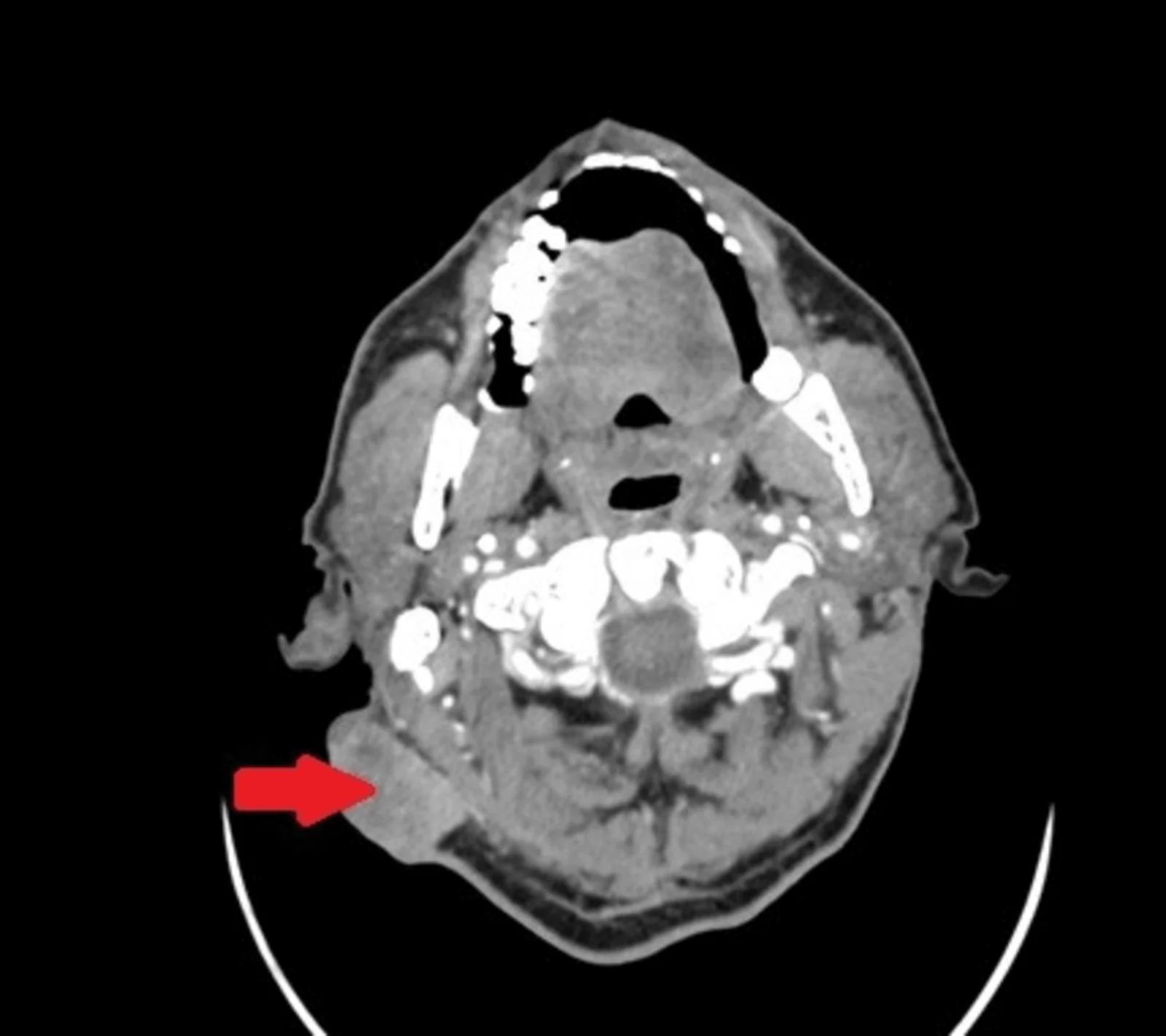
Contrast-enhanced CT scan demonstrating a well-defined, enhancing subcutaneous lesion in the right postauricular region (red arrow) without muscular or bony invasion.

CT of the neck demonstrated a well-defined, irregular soft tissue lesion measuring approximately 38 × 21 × 25 mm involving the subcutaneous tissue of the right postauricular region. Mild heterogeneous contrast enhancement was observed without extension into the muscular plane or underlying bony erosion. No cervical lymphadenopathy was identified (Figure 2).

**Figure 3:**
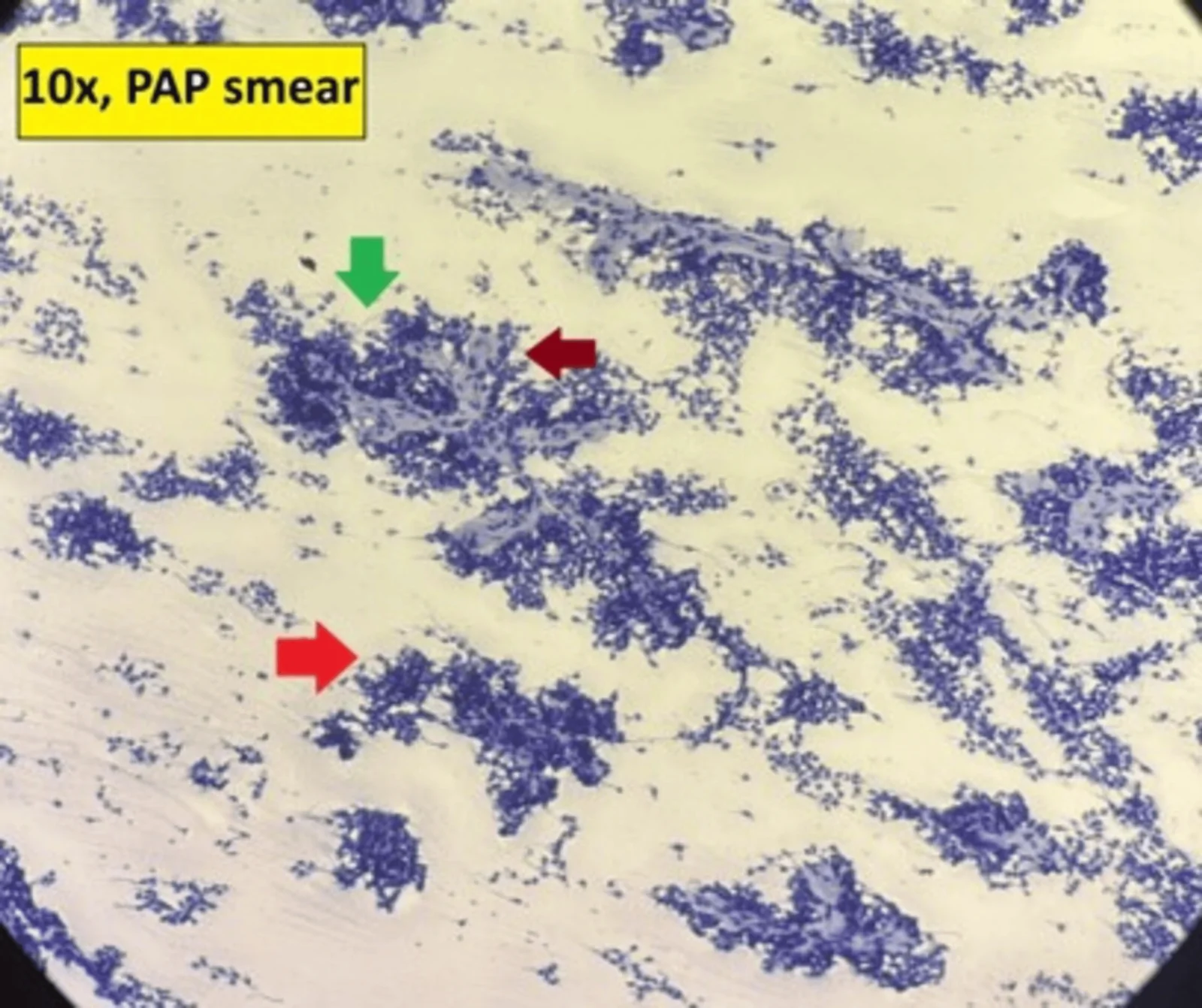
Cytology smear showing basaloid to ovoid tumor cells arranged in tight clusters (red arrow), a papillary pattern (green arrow) with hyaline stromal cores (maroon arrow), and singly scattered cells.

Fine-needle aspiration cytology revealed a highly cellular lesion composed of basaloid to ovoid cells arranged in clusters and papillary fragments with hyaline stromal cores, favoring a benign adnexal tumor (Figure 3).

**Figure 4:**
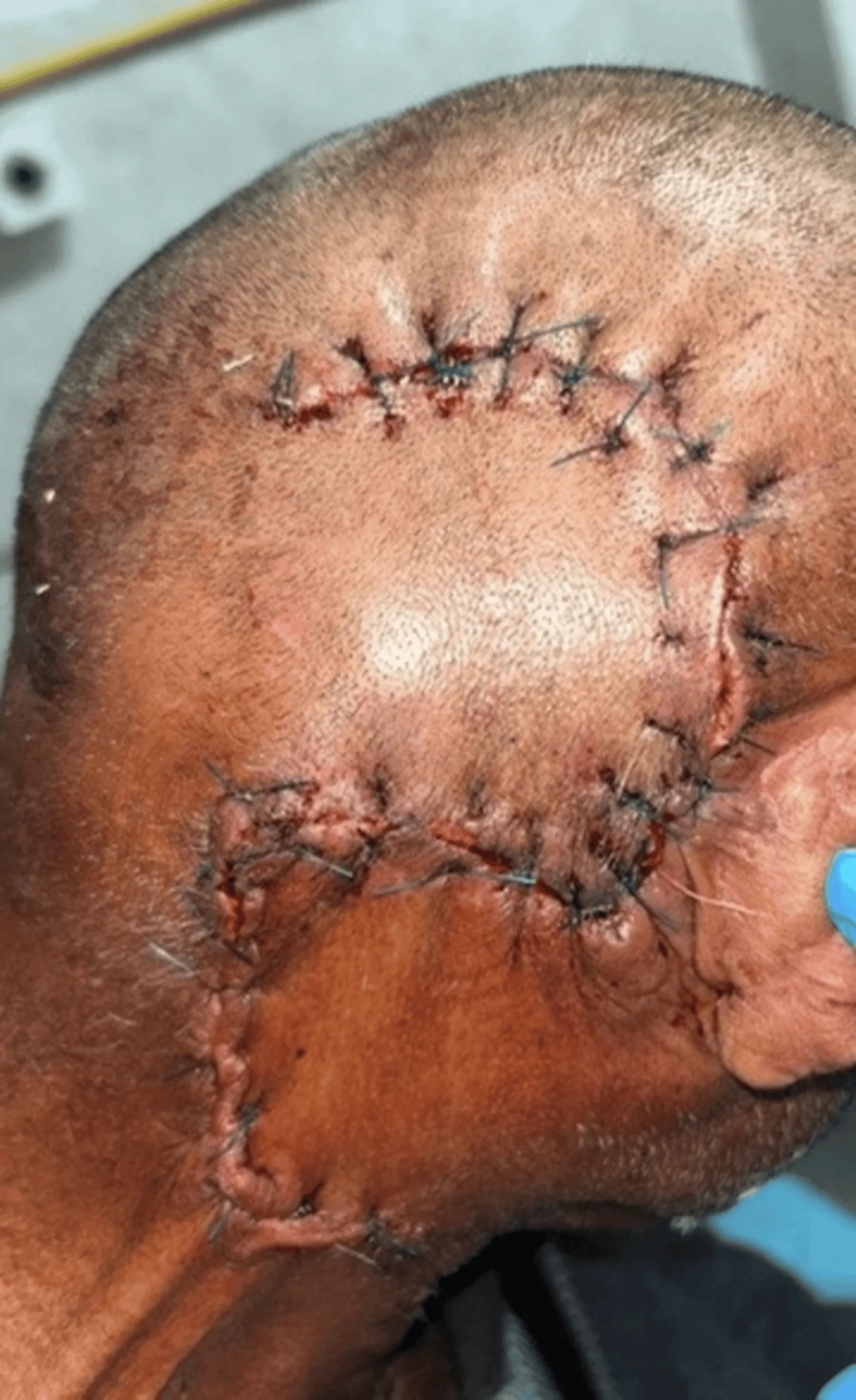
Immediate postoperative photograph demonstrating complete excision of the lesion with local flap reconstruction.

The patient subsequently underwent complete surgical excision of the lesion with adequate margins, followed by reconstruction using a local postauricular flap. The postoperative recovery was uneventful, without wound complications (Figure 4).

**Figure 5:**
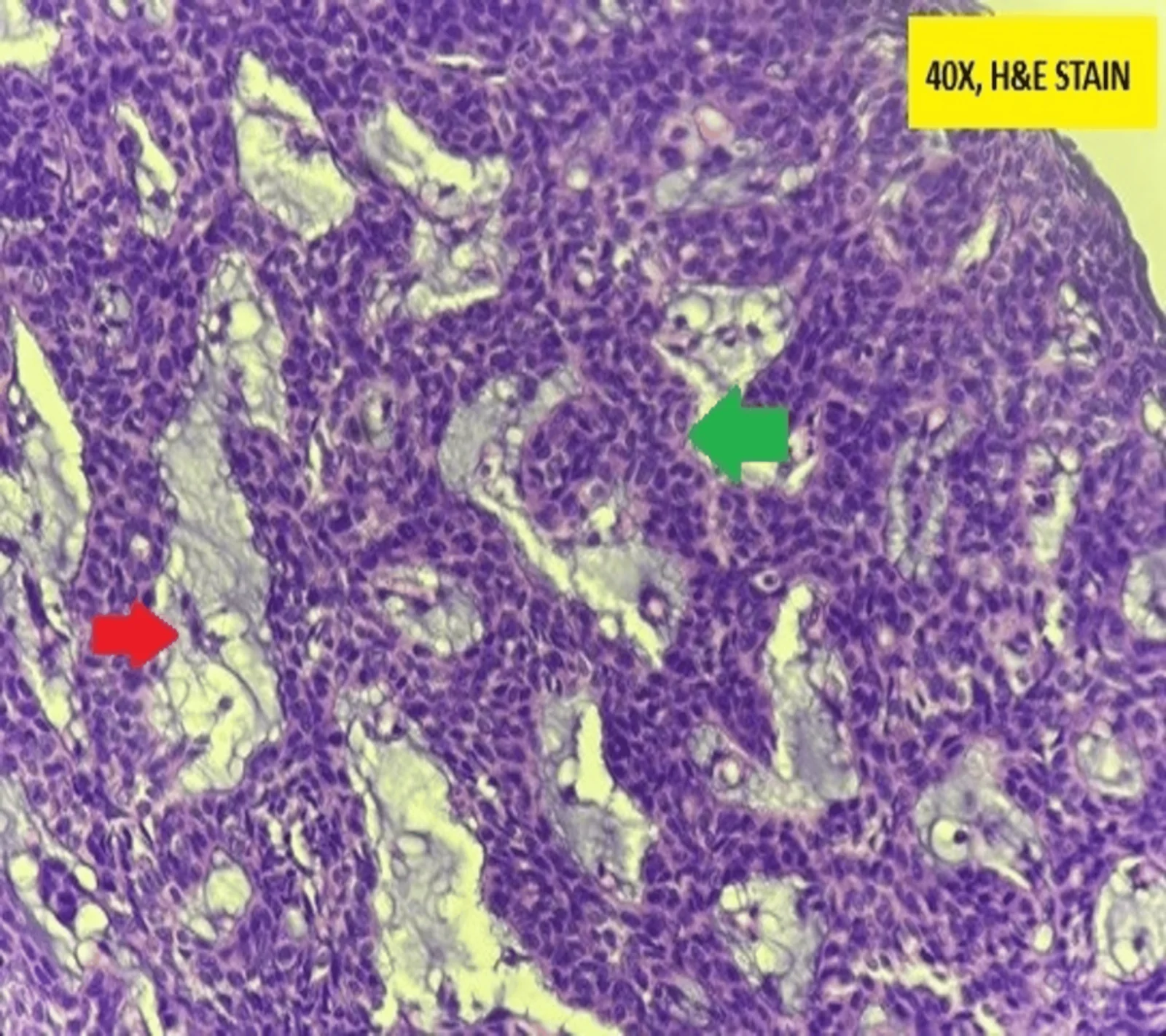
Histopathological section (H&E stain, 40×) demonstrating solid nests (green arrow), microcystic architecture (red arrow), and a dual cell population characteristic of atypical nodular hidradenoma.

Gross examination of the excised specimen showed a skin-covered, encapsulated lesion measuring 7 × 5.2 × 1.5 cm. Histopathological examination demonstrated a well-circumscribed dermal tumor composed of solid nests, cribriform areas, and microcystic spaces. The lesion exhibited a dual cell population consisting of polygonal eosinophilic cells and clear myoepithelial-like cells with intervening fibro-myxoid to hyalinized stroma. No tumor necrosis, atypical mitotic figures, lymphovascular invasion, or perineural invasion was identified. Although the margins were predominantly pushing, focal permeative borders were noted (Figure 5).

**Figure 6:**
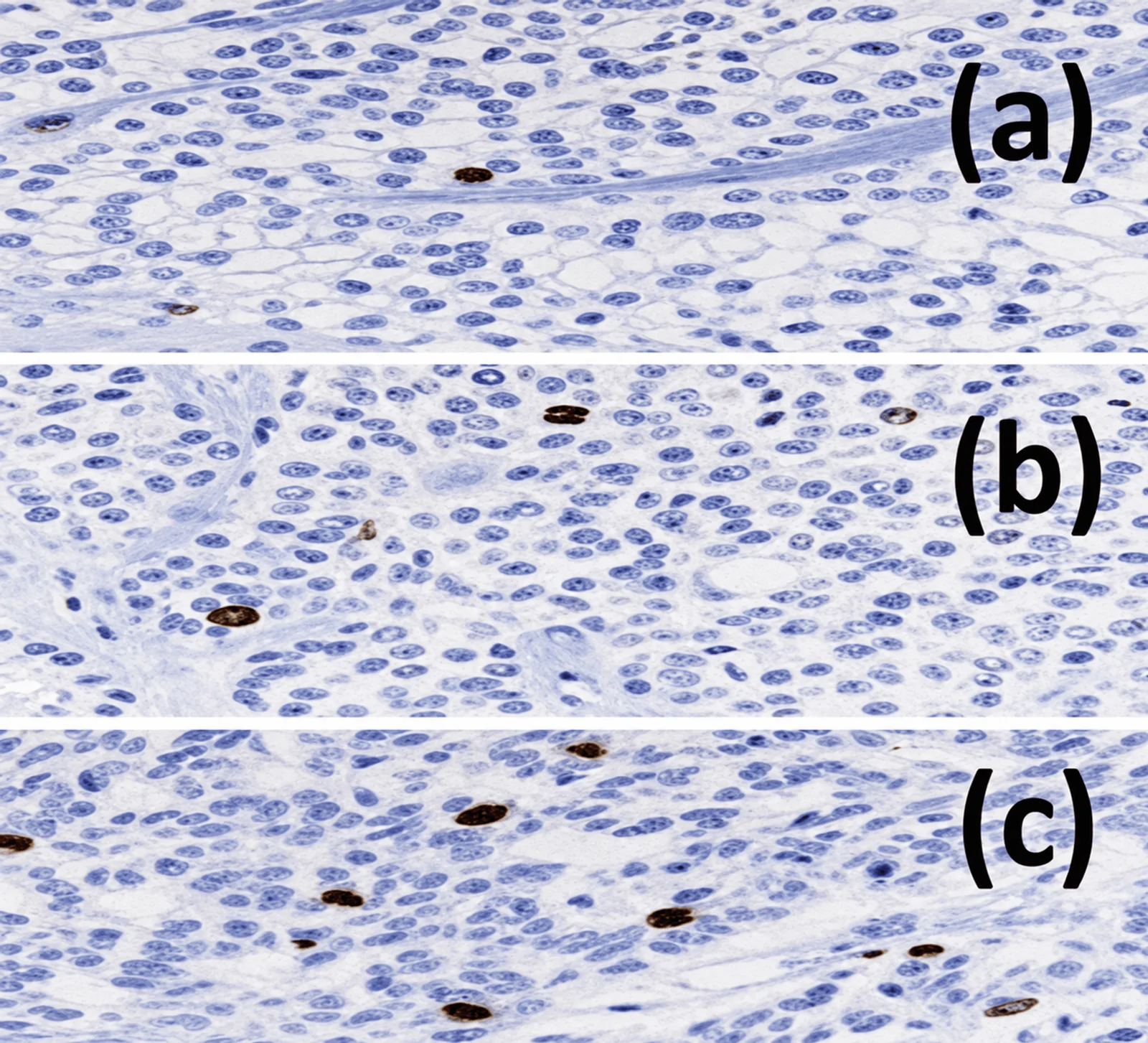
Immunohistochemical staining demonstrating a low Ki-67 (a) proliferation index with focal SMA positivity (b) and patchy S100 positivity (c).

Because of the atypical morphology, immunohistochemistry was performed. Tumor cells showed a Ki-67 labeling index of approximately 1%, focal smooth muscle actin positivity, patchy S100 positivity, and negative p63 staining. These findings confirmed the diagnosis of atypical nodular hidradenoma (Figure 6a-6c).

At six-month follow-up, the surgical wound had healed satisfactorily, with no evidence of local recurrence.

## Discussion

Nodular hidradenoma is an uncommon benign sweat gland tumor, accounting for a small proportion of cutaneous adnexal neoplasms. The majority arise on the trunk and extremities, whereas postauricular involvement is exceedingly uncommon, making clinical diagnosis difficult [4]. Patients typically present with a slowly enlarging, painless mass that may clinically resemble epidermoid cysts, dermoid cysts, proliferating pilar tumors, schwannomas, or other benign soft tissue tumors.

Radiological evaluation helps determine lesion extent but lacks specific imaging characteristics for definitive diagnosis. In the present case, CT demonstrated a well-defined, enhancing subcutaneous lesion without invasion of adjacent structures, findings consistent with previously reported benign adnexal tumors.

Histologically, atypical nodular hidradenoma occupies an intermediate position between benign hidradenoma and hidradenocarcinoma. The differential diagnoses include post-traumatic schwannoma (a slow-growing tumor of Schwann cells arising after trauma, surgery, or chronic irritation of a nerve), low-grade fibromyxoid sarcoma (rare in the head and neck region and common in the deep soft tissues of the thigh, trunk, and shoulder), dermoid cyst (benign congenital masses formed from trapped skin tissue, hair, oil glands, or even teeth, often appearing as painless, slow-growing, soft-to-doughy, nontender, well-circumscribed bumps on the scalp, face, or neck at birth or in childhood), and epidermal inclusion cyst (benign, slow-growing skin lumps filled with keratin, often appearing on the face, scalp, or neck, sometimes with a central blackhead-like punctum). Features such as focal infiltrative borders, increased architectural complexity, and cytological atypia raise concern for aggressive behavior; however, the absence of significant mitotic activity, tumor necrosis, vascular invasion, and a high proliferative index argue against malignancy [5,6].

Immunohistochemistry plays an important role in distinguishing hidradenoma from its histological mimics. In benign hidradenomas, the Ki-67 index is typically low (<5-10%). A Ki-67 index greater than 10-11% strongly suggests malignant potential (hidradenocarcinoma) or an atypical variant requiring close monitoring. The low Ki-67 index observed in our patient supports the relatively indolent biological behavior of the lesion. Patchy S100 positivity and focal SMA positivity indicate myoepithelial differentiation, whereas negative p63 staining helped exclude other adnexal neoplasms [7,8].

The principal treatment is complete surgical excision with histologically negative margins. Recurrence has been reported following incomplete excision, emphasizing the importance of meticulous surgical clearance. Although malignant transformation is rare, long-term surveillance remains advisable because the biological behavior of atypical nodular hidradenoma has not been completely elucidated [5,9].

Our case illustrates the importance of correlating clinical findings, radiological imaging, histopathology, and immunohistochemistry in establishing the diagnosis of this rare tumor and differentiating it from other cutaneous adnexal lesions.

## Conclusions

Atypical nodular hidradenoma is a rare adnexal neoplasm that may clinically mimic several benign and malignant lesions of the head and neck. Imaging findings are nonspecific, and definitive diagnosis relies on histopathological examination with immunohistochemical confirmation. Complete surgical excision remains the treatment of choice and is associated with an excellent prognosis when adequate margins are achieved. Owing to the possibility of local recurrence, periodic follow-up is recommended.
